# Behavioral and psychological symptoms in dementia is not a unitary concept: A critical review with emphasis on Alzheimer’s disease

**DOI:** 10.1590/S1980-57642009DN20400007

**Published:** 2008

**Authors:** Jerson Laks, Eliasz Engelhardt

**Affiliations:** 1 Center for Alzheimer’s Disease/ Institute of Psychiatry/Federal University of Rio de Janeiro; School of Medical Sciences, State University of Rio de Janeiro.; 2 Institute of Neurology Deolindo Couto of the Federal University of Rio de Janeiro.

**Keywords:** BPSD, dementia, psychosis, depression, apathy, elderly

## Abstract

Behavioral and Psychological Symptoms of Dementia (BPSD) is an important feature of dementia. However, this definition comprises a large array of symptoms and syndromes. This hampers understanding of the behavior of patients with dementia and the devising of strategies to ameliorate these symptoms.

**Objectives:**

This review aimed to describe the main factors and syndromes that comprise BPSD, as well as neuroimaging, psychopharmacological, and genetic data derived from studies of these factors.

**Methods:**

A search on the Medline, Scielo, and ISI databases was performed using the keyword BPSD for articles published within the last five years. Selected publications were favored, so this review should not be regarded as a systematic study on the subject.

**Results:**

The main factors and syndromes comprising BPSD were identified, namely psychosis, depression, and activity. Different ways of clustering symptoms were considered. The main manifestations of psychosis, apathy and depression were focused, relating phenomenology to neuroimaging and pharmacological issues.

**Conclusions:**

BPSD is a heterogeneous array of symptoms which can be better understood as clusters. At least three factors can be separated in BSPD, namely psychosis, depression, and activity. This division may offer guidance to clinicians regarding treatment management and follow up of the chosen therapeutic strategy.

## Abstract (pt)

Os Sintomas Psicológicos e do Comportamento na Demência (SPCD) são uma característica importante das demências. Entretanto, essa definição congrega uma ampla gama de sintomas e síndromes. Essa é uma fonte de problemas no que tange o melhor conhecimento do comportamento dos pacientes e daí o desenvolvimento de estratégias para aliviar esses sintomas.

**Objetivos:**

Essa revisão objetiva descrever os principais fatores e síndromes que compõem os SPCD, além de alguns dados de neuroimagem, psicofarmacológicos e genéticos derivados do estudo desses fatores.

**Métodos:**

Foi realizada busca nos bancos de dados Medline, Scielo e ISI utilizando a palavra chave BPSD (SPCD) compreendendo os cinco últimos anos. Foram privilegiadas publicações selecionadas, de modo que a presente revisão não deve ser vista como do tipo sistemática sobre o assunto.

**Resultados:**

Os principais fatores e síndromes que compõem os SPCD foram identificados, compreendendo psicose, depresão e atividade. São consideradas diferentes maneiras de se conglomerar os sintomas. As manifestações principais, psicose, apatia e depressão são focalizados, relacionando a fenomenologia à aspectos de neuroimagem e farmacológicos.

**Conclusões:**

Os SPCD constituem um conjunto heterogêneo de sintomas que podem ser melhor compreendidos como conglomerados. Ao menos três fatores podem ser separados nos SPCD, psicose, depressão e atividade. Essa divisão pode oferecer uma melhor orientação para o clínico visando o tratamento e o seguimento da resposta a uma determinada estratégia terapêutica escolhida.

Behavioral and Psychological Symptoms in Dementia (BPSD)^1^ is a syndrome first proposed a decade ago. It was promptly recognized as an important constituent of dementia, along with the cognitive decline and impairment in activities of daily living. These symptoms include agitation, depression, apathy, delusions, hallucinations, physical and verbal aggression, wandering, sexual disinhibition, anxiety, irritability, euphoria, and eating and sleep disturbances. Several studies have consistently confirmed the high prevalence of BPSD in clinical settings,^1^ nursing homes,^2^ and among community-dwelling elderly.^3^ Overall, prevalence rates of BPSD range from 60% to 90% of the elderly with dementia. These symptoms represent an important factor in increasing costs of dementia care, and contribute to caregiver distress and burnout.^4-6^

Despite the increasing number of studies covering subjects that deal with various aspects of BSPD, from clinical characterization to different treatment strategies, there is still a lively debate on each of these issues. Firstly, the clinical description of BPSD comprises an array of symptoms and syndromes that are sometimes difficult to separate from one another on clinical grounds. Depression, anxiety, agitation, and disinhibition commonly occur together in the same patient, making it difficult for health professionals to decide which syndrome should be focused with regard to establishing a treatment strategy. Furthermore, the symptoms tend to be unstable, lasting for limited and uncertain periods of time. This poses an extra problem regarding the best way clinicians and researchers should evaluate symptoms. It seems that defining recent periods of time and the rating of setting-specific symptoms might be the best approach.^7^ In fact, at least one study has proved this to be the best approach, showing it to be more sensitive to treatment effects than having caregivers alone rate symptoms.^8^

Pharmacological treatments depend on the proper definition of the main syndrome, and not achieving good operational criteria may result in failure to design sound treatment strategies, rather than indicating inefficacy of the drugs. The quantification of symptoms depends on the scales used, and this may be a relevant source of difference among studies. Scales of aggression and agitation, and scales for depression may be more specific than general scales for BPSD. However, these more specific scales may yield data that are in fact an integral part of the more general status of patients, raising doubts about the accuracy of the diagnosis. Another important issue is related to the size effect of the treatments for BPSD and the clinical relevance of some statistically significant results in double-blind placebo controlled studies, which again may be dependent on the target symptoms which are being studied.^9-11^

The increasing wealth of literature on BPSD permits the presentation of some organized thoughts on the issue. The aim of this structured review was to describe the main subset of BPSD clusters and to show some studies that have been able to associate clinical profiles with neuroimaging, neurobiological, and psychopharmacological data.

## Methods

A search on the Medline, Scielo, and ISI databases was performed using the keyword BPSD for articles published over the last five years. After checking for cross references, the available abstracts were read by one of the authors (JL) and publications then selected according to their relevance to the issue. For the sake of brevity and limited space, the authors decided to concentrate on just some of the publications, and so this review should not be regarded as a systematic review on the subject.

### BPSD clusters: does this help us to better recognize the syndromes?

As previously mentioned, the broad definition of BPSD may be elusive about what kind of symptomatology the patient is really experiencing. Some groupings of BPSD symptoms have been proposed so as to better define the clinical cases and to allow some correlation with possible neurobiological causes. With regard to the underlying neurobiological factors, the research is sometimes hampered by the small number of patients and by the problems in definitions of the symptoms to be studied. Thus, the generalization of these findings should be done with caution, given the methodological problems defined above.

The most common grouping is condensed into three clusters, namely the depressive, psychotic and overactive “factors”.^12^ However, a recent study by a European consensus found that psychosis, psychomotor factor, mood liability factor, and instinctual factor form four distinct behavioral syndromes in dementia. The psychosis factor comprises irritability, agitation, hallucinations and anxiety. The psychomotor factor is constituted by aberrant motor behavior and delusions, whereas the mood liability factor and the instinctual factor are formed by disinhibition, elation and depression, appetite disturbance, sleep disorders and apathy, respectively.^13^ The same group of researchers summarized data showing that these BPSD clusters are related to some biological features.^14^ Similarly, another study with a large population sample, devised four components of BPSD: behavioral dysfunction (euphoria, disinhibition, aberrant motor behavior, and sleep and appetite disturbances), psychosis (delusions and hallucinations), mood (depression, anxiety, and apathy), and agitation (aggression and irritability). Higher behavioral dysfunction, agitation, and mood component scores were associated with lower age at onset. Behavioral dysfunction and mood component scores were associated with gender. However, none of the components were associated with age at assessment, years of education, or number of APOE ε4 alleles.^15^

A development subsequent to the recognition of three to four clusters in BPSD, was the proposal of two distinct diagnoses besides BPSD, defined as the Psychosis of Alzheimer’s Disease^16^ (AD) and the Alzheimer Associated Psychotic Disorder,^17^ and the Depression of AD,^18^ all of them with provisional criteria as candidates to join the DSM-V and International Classification Systems.

### Psychosis

Psychosis of AD has an operational definition with all signs and symptoms resembling psychotic syndrome in adults, with the condition that it has to have started only after the onset of the cognitive and functional impairment typical of AD.^16^ The patient must have no history of previous psychiatric disorders, and a confirmed diagnosis of probable AD according to the DSM-IV and the NINCDS-ADRDA criteria.

Overall, the role of the diseased frontal lobes in inducing behavioral dysfunctions, with increased agitation and delusions, is more evident in the Frontotemporal dementia complex. However, one should bear in mind that in these cases that agitation and psychosis in AD is also likely to be a consequence of more pronounced pathology involving the frontal lobes.^19^

Psychosis of AD is related to frontal and temporal hypoperfusion on SPECT and to lower levels of N-acetylaspartate and increased myo-inositol in the anterior cyngulate gyrus. Delusions and activity disturbance were shown to be significantly correlated with alterations in the anterior cingulate gyrus, but not in the posterior cyngulate, in patients with BPSD compared to AD patients without BPSD, whereas cognitive and functional impairments were correlated to lower posterior cyngulate levels of N-Acetylaspartate.^20^ Another recent study found differences in frontal perfusion with SPECT related to gender in AD patients with or without BPSD. Female patients with psychosis presented lower perfusion in right inferolateral prefrontal cortex and in inferior temporal regions compared to female patients without such symptoms. Conversely, male patients with psychotic symptoms showed higher perfusion in the right striatum compared to male patients without these symptoms. It is reasonable to hypothesize that the right hemisphere prefrontal and lateral temporal cortex play a role in the psychosis of AD in women but not in men.^21^

Neurotransmitters may also show imbalances and changes in concentration at certain sites that help to explain the symptoms, rather than just the observed widespread neurodegeneration. While the basal forebrain degeneration of the cholinergic pathways is a hallmark of Alzheimer’s disease, a study with post mortem measurement of cholinergic and serotonergic concentrations has found that the imbalance between these two systems accounted for the emergence of BPSD, mainly psychosis.^22^ A marked depletion occurred in serotonin and in its metabolite levels both in frontal and temporal cortex of AD patients, which did not correlate with cognitive symptoms. However, the cognitive impairment correlated with the cholinergic/serotonergic ratio, consistent with the hypothesis that 5-HT seems to act as an inhibitory neurotransmitter on cholinergic neurons. Therefore, decreases in the serotonergic tone would help to maintain the cholinergic input in deficient target areas where this neurotransmitter is needed.^23^ Moreover, the lower serotonergic levels in the frontal lobe predicted the overactivity factor of BPSD while lower levels of serotonin in the temporal lobe predicted psychosis, again more frequently occurring in women.

Another source of relevant information toward confirming the hypothesis that the susceptibility factor for BPSD also has a genetic contribution involves gathering data on genes encoding components of the serotonergic system.^24^ A consequence of this approach is the possibility to develop specific treatment targets using drugs according to genetic profiles. A large prospective cohort of patients with Alzheimer’s disease was studied regarding the *5HT2A* T102C and *5HT2C* cys23ser genotype along with allele frequencies and their correlations with BPSD. An increased frequency of the C allele and CC genotype of the T102C variant of *5HT2A* correlated with hallucinations, delusions, psychosis and aberrant motor behavior. Moreover, the C allele and CC genotype frequencies of the cys23ser variant of *5HT2C* was also shown, for the first time, to correlate with anxiety in females.^24^

### Apathy and depression

There is no reason to think that only one neurotransmitter system would be responsible for each cluster of BPSD, but it may well be that a complex interaction of many neurotransmitters and neuroreceptors play a role to make patients express certain symptoms. The role of γ-aminobutyric acid (GABA) and of the glutamatergic (NMDA) receptors in BPSD is of interest since they are the major inhibitory and excitatory neurotransmitters of the Central Nervous System, respectively. Some animal and human studies suggest that anxiety, psychotic symptoms, aggression, and depression are related to GABAergic dysfunction,^25-26^ whereas the NMDA receptors and glutamatergic dysfunctions have been well documented in AD^27^. A study found a positive relationship between apathy and depression, and increased plasma levels of GABA in 14 patients at severe stages of dementia.^28^ This may also be in agreement with a disruption of the glutamatergic system during severe stages of the disease, revealing an imbalance that might be amenable to certain pharmacologic strategies tailored for specific BPSD clusters. In another study, post mortem brains and clinical data from 43 patients with dementia were compared to a matched sample of 24 normal elderly.^29^ Similarly, significant loss of GABA content with no changes in glutamate levels were found in the cerebral cortex of AD patients. Presynaptic GABAergic system GABA concentration and GABA/glutamate ratio were negatively correlated to depression factor while GABA_A_ agonist binding site *Bmax* and BZ site *Bmax* were positively correlated with the depression factor. In other words, a complex interaction between presynaptic and postsynaptic disruptions of the GABAergic system plays a role in depression but not in cognitive decline in AD patients.

A recent review on the phenomenology, neuroimaging and neurobiological profiles of apathy in dementia, mainly in AD, proposed that apathy may be the result of a dysfunction in affective-emotional pro­cessing which takes place in ventromedial prefrontal cortex and orbitofrontal cortex, and their connections with amygdala and nucleus accum­bens, leading to impairment in striatum dopaminergic ac­tivation.^30^ Several studies have concurred with data confirming this proposition, although the whole concept may be difficult to prove. Nevertheless, such a concept may lead to some treatment strategies in certain diseases which involve the basal ganglia and the frontal cortex, such as Parkinson’s disease and related disorders, as well as Alzheimer’s disease.

Depression of AD is a provisional diagnosis currently being studied and which is best evaluated with the Cornell Depression in Dementia Scale.^31^ The proposed criteria for Depression of Alzheimer’s disease comprise those of the DSM-IV for depression and include the additional symptoms of irritability and social isolation/withdrawal.^18^

Depression is a syndrome amenable to treatment. The study of the possible mechanisms of depression is of utmost importance in order to provide a better course with fewer complications in the outcome of patients with dementia. Some of the studies mentioned above show that depression is related to some of the same neuropathological, psychopharmacological, and functional problems that result in apathy.^11,17,22-24^ However, a large sample of nursing home elderly in Norway has shown that depression can also be subdivided into 4 categories according to score on the Cornell Scale^32^ These 4 factors (mood, cyclic, physical, and behavioral) may be phenomenological presentations of the depressive spectrum, and it is also conceivable that they represent certain complementary pathophysiological and neuropsychopharmacological pathways.

As previously mentioned, the involvement of specific regions of the frontal lobe is liable to promote some BPSD sub-syndromes, such as depression, psychosis, or apathy. The neurodegeneration observed in the brain of Alzheimer’s disease patients is correlated with cognitive symptoms, and some studies have demonstrated that the tau, phospho-tau and beta-amyloid concentrations in Central Spinal Fluid of these patients correlate only with apathy, but not with psychosis and depression.^33^ In Dementia with Lewy bodies, psychosis is considered to be a core symptom of the disease and not an integral part of BPSD in dementia.^34^ However, investigating other BPSD sub-syndromes in these patients is also important given that some of them may be key factors causing further agitation and impairments that affect quality of life both in patients and caregivers. A study showed that anxiety, depression, and apathy were highly frequent in a sample of patients with Dementia with Lewy bodies, whereas psychosis was present in 50% of them.^35^ The symptoms did not correlate with severity of disease or of motor impairment, which may be a sign that distinct lesions in certain regions of the brain give rise to this symptomatology. These data highlight the importance of searching beyond psychosis in Dementia with Lewy bodies.

## Conclusion

BPSD encompasses a heterogeneous array of symptoms, but these can be better understood as clusters. These features warrant future study to better describe the phenomenology and their relationship with the neurohistopathological lesions, neuroimaging findings, and psychopharmacological data presented to date. At least three factors can be separated in BSPD, namely psychosis, depression, and activity factors. This division may offer better guidance for clinicians on treatment strategies and follow up of the response to the chosen therapeutic strategy. Although neuroimaging, genetic, and psychopharmacological studies are not yet able to provide definitive answers on the causality of symptom presentation, there is a growing body of literature seeking further data and confirming the evidence found so far.
